# Supplementary material for: Extent and reproduction of coastal species on plastic debris in the North Pacific Subtropical Gyre
Source: Nat Ecol Evol. 2023 Apr 17;7(5):687–97. doi: 10.1038/s41559-023-01997-y (PMC10172146; doi:10.1038/s41559-023-01997-y)
Supplement: Supplementary file 1 — Supplementary Tables 1 and 2. [file 41559_2023_1997_MOESM1_ESM.pdf]

nature ecology & evolution

Article

https://doi.org/10.1038/s41559-023-01997-y

Extent and reproduction of coastal species on plastic debris in the North Pacific Subtropical Gyre

In the format provided by the authors and unedited

Supplementary Table 1. Frequency (% of biofouled debris) and life history characteristics of observed coastal and pelagic taxa.

Column labels are defined as follows: Phylum = phylum name; Taxon = species or taxon name; Frequency = percentage of biofouled debris on which the taxon was observed; Mobility (sessile or mobile); Reproduction (sexual or asexual); Fertilization (internal or external); Development (direct, planktonic = lecithotrophic or planktotrophic planktonic); Trophic Level (omnivore, detritivore, herbivore, carnivore); Feeding Mechanism (surface feeder, deposit feeder, filter feeder, suspension feeder, predator).

Phylum	Taxon	Frequency	Adult Mobility	Reproduction	Fertilization	Larval Development	Trophic Position	Feeding Mechanism	References
**Coastal Taxa**									
Arthropoda	*Stenothoe gallensis*	36.90%	Mobile	Sexual	Internal	Direct	Omnivore	Grazer	JTMD NEMESIS [42]; Ambrose and Anderson 1990 [67]
Cnidaria	*Aglaophenia* aff. *pluma*	25.20%	Sessile	Sexual/Asexual	Internal	Planktonic	Omnivore	Suspension feeder	Svoboda & Cornelius 1991 [68]; Choong et al. 2018 [30]
Arthropoda	*Ianiropsis serricaudis*	24.30%	Mobile	Sexual	Internal	Direct	Omnivore	Grazer	JTMD NEMESIS [42]
Arthropoda	*Calliopius pacificus*	19.40%	Mobile	Sexual	Internal	Direct	Carnivore	Predator	Macdonald et al. 2010 [69]
Bryozoa	*Aetea* sp. A	17.50%	Sessile	Sexual/Asexual	Internal	Planktonic	Omnivore	Suspension feeder	JTMD NEMESIS [AETEA] [42]; Bock 1982 [BRYOZOA] [70]; Weaver et al. 2018 [BRYOZOA] [71]
Arthropoda	*Elasmopus rapax*	14.60%	Mobile	Sexual	Internal	Direct	Detritivore	Grazer	Ambrose and Anderson 1990 [67]; Ferreira et al. 2019 [72]
Cnidaria	*Anthopleura* sp. A	12.60%	Sessile	Sexual/Asexual	Internal/External	Planktonic	Carnivore	Suspension feeder/Predator	Bocharova & Kozevich 2011 [ANTHOZOA] [73]
Cnidaria	*Anthopleura* sp. B	8.70%	Sessile	Sexual/Asexual	Internal/External	Planktonic	Carnivore	Suspension feeder/Predator	Bocharova & Kozevich 2011 [ANTHOZOA] [73]
Cnidaria	*Diadumene lineata*	7.80%	Sessile	Sexual/Asexual	External	Planktonic	Carnivore	Suspension feeder/Predator	JTMD NEMESIS [42]
Annelida	Spirorbidae sp.	7.80%	Sessile	Sexual	Internal	Planktonic	Omnivore	Suspension feeder	Terlizzi et al. 2000 [74]
Cnidaria	*Anemonia erythraea*	5.80%	Sessile	Sexual/Asexual	Internal/External	Planktonic	Carnivore	Suspension feeder/Predator	Bocharova & Kozevich 2011 [ANTHOZOA] [73]
Bryozoa	*Scruparia ambigua*	3.90%	Sessile	Sexual/Asexual	Internal	Planktonic	Omnivore	Suspension feeder	Bock 1982 [BRYOZOA] [70]; Weaver et al. 2018 [BRYOZOA] [71]
Cnidaria	*Clytia hemisphaerica*	2.90%	Sessile	Sexual/Asexual	External	Planktonic	Omnivore	Suspension feeder	Cornelius 1982 [29]; Choong et al. 2018 [30]; Takeda et al. 2018 [75]
Arthropoda	*Endeis nodosa*	2.90%	Sessile	Sexual	Internal	Benthic	Carnivore	Predator	JTMD NEMESIS [42]
Porifera	*?Haliclona* sp.	2.90%	Sessile	Sexual/Asexual	Internal/External	Planktonic	Omnivore	Filter feeder	Maldodano & Riesgo 2008 [PORIFERA] [76]
Annelida	*Hydroides* sp. cf. *ezoensis*	2.90%	Sessile	Sexual	External	Planktonic	Omnivore	Suspension feeder	JTMD NEMESIS [42]
Cnidaria	*Anthopleura* sp. D	1.90%	Sessile	Sexual/Asexual	Internal/External	Planktonic	Carnivore	Suspension feeder/Predator	Bocharova & Kozevich 2011 [ANTHOZOA] [73]
Bryozoa	*Catenicella* sp.	1.90%	Sessile	Sexual/Asexual	Internal	Planktonic	Herbivore	Suspension feeder	Bock 1982 [BRYOZOA] [70]; Weaver et al. 2018 [BRYOZOA] [71]
Bryozoa	*Crisia* sp.	1.90%	Sessile	Sexual/Asexual	Internal	Planktonic	Herbivore	Suspension feeder	Bock 1982 [BRYOZOA] [70]; Weaver et al. 2018 [BRYOZOA] [71]
Bryozoa	*Disporella* sp.	1.90%	Sessile	Sexual/Asexual	Internal	Planktonic	Herbivore	Suspension feeder	Bock 1982 [BRYOZOA] [70]; Weaver et al. 2018 [BRYOZOA] [71]
Arthropoda	*Jassa marmorata*	1.90%	Mobile	Sexual	Internal	Direct	Omnivore	Suspension feeder/Predator	JTMD NEMESIS [42]
Cnidaria	*Plumularia strictocarpa*	1.90%	Sessile	Sexual/Asexual	Internal	Planktonic	Omnivore	Suspension feeder/Predator	Hirohito 1995 [77]; Kolzoff 1990 [PLUMULARIA] [79]; JTMD NEMESIS [PLUMULARIA] [42]
Annelida	Nereididae sp.	1.90%	Mobile	Sexual	External	Planktonic	Omnivore	Grazer/Predator	JTMD NEMESIS [NEREIDIDAE] [42]
Bryozoa	*?Tubulipora* sp	1.90%	Sessile	Sexual/Asexual	Internal	Planktonic	Omnivore	Suspension feeder	JTMD NEMESIS [42]; Bock 1982 [BRYOZOA] [70]; Weaver et al. 2018 [BRYOZOA] [71]
Bryozoa	*Aetea anguina* ?	1.00%	Sessile	Sexual/Asexual	Internal	Planktonic	Omnivore	Suspension feeder	JTMD NEMESIS [AETEA] [42]
Bryozoa	*Aetea* sp. B	1.00%	Sessile	Sexual/Asexual	Internal	Planktonic	Omnivore	Suspension feeder	JTMD NEMESIS [AETEA] [42]
Bryozoa	*Amathia gracilis*	1.00%	Sessile	Sexual/Asexual	Internal	Planktonic	Herbivore	Suspension feeder	Bock 1982 [BRYOZOA] [70]; Weaver et al. 2018 [BRYOZOA] [71]; Reed 1988 [79]
Arthropoda	Amphilochidae sp.	1.00%	Mobile	Sexual	Internal	Direct	Omnivore	Grazer/Predator	Guerra-García et al. 2014 [AMPHILOCHIDAE] [80]
Cnidaria	*Antennella secundaria*	1.00%	Sessile	Sexual/Asexual	Internal	Direct	Omnivore	Suspension feeder	Hirohito 1995 [77]
Bryozoa	*Bugula tsunamiensis*	1.00%	Sessile	Sexual/Asexual	Internal	Planktonic	Herbivore	Suspension feeder	Bock 1982 [BRYOZOA] [70]; Weaver et al. 2018 [BRYOZOA] [71]
Bryozoa	*Callaetea* sp.	1.00%	Sessile	Sexual/Asexual	Internal	Planktonic	Herbivore	Suspension feeder	Bock 1982 [BRYOZOA] [70]; Weaver et al. 2018 [BRYOZOA] [71]
Mollusca	*Crassostrea gigas*	1.00%	Sessile	Sexual	External	Planktonic	Omnivore	Filter feeder	JTMD NEMESIS [42]
Bryozoa	*Cryptosula pallasiana*	1.00%	Sessile	Sexual/Asexual	Internal	Planktonic	Omnivore	Suspension feeder	JTMD NEMESIS [CRYPTOSULA] [42]
Mollusca	*Musculus cupreus*	1.00%	Sessile	Sexual	Internal	Direct	Omnivore	Filter feeder	JTMD NEMESIS [42]
Porifera	*?Leucosolenia* sp.	1.00%	Sessile	Sexual/Asexual	Internal/External	Planktonic	Omnivore	Filter feeder	Maldodano & Riesgo 2008 [PORIFERA] [76]
Porifera	*?Sycon* sp.	1.00%	Sessile	Sexual/Asexual	Internal/External	Planktonic	Omnivore	Filter feeder	Maldodano & Riesgo 2008 [PORIFERA] [76]
Porifera	*?Halichondria* sp.	1.00%	Sessile	Sexual/Asexual	Internal/External	Planktonic	Omnivore	Filter feeder	Maldodano & Riesgo 2008 [PORIFERA] [76]
**Pelagic Taxa**									
Bryozoa	*Jellyella* spp.	75.70%	Sessile	Sexual/Asexual	Internal	Planktonic	Omnivore	Suspension feeder	Bock 1982 [BRYOZOA] [70]; Weaver et al. 2018 [BRYOZOA] [71]; Taylor & Monks 1997 [81]
Arthropoda	*Lepas* spp.	65.00%	Sessile	Sexual	Internal	Planktonic	Omnivore	Suspension feeder/Predator	Howard & Scott 1959 [82]; Patel 1959 [83]; Bieri 1966 [84]; Moyse 1987 [85]
Arthropoda	*Planes* spp.	40.80%	Mobile	Sexual	Internal	Planktonic	Omnivore	Grazer/Predator	Frick et al. 2011 [44]
Arthropoda	*Caprella andreae*	25.20%	Mobile	Sexual	Internal	Direct	Omnivore	Grazer/Predator	JTMD NEMESIS [CAPRELLA] [42]
Cnidaria	*Obelia griffini*	25.20%	Sessile	Sexual/Asexual	Internal	Planktonic	Omnivore	Suspension feeder	JTMD NEMESIS [42]
Arthropoda	*Plagusia* sp.	7.80%	Mobile	Sexual	Internal	Planktonic	Omnivore	Grazer/Predator	Frick et al. 2011 [44]
Annelida	*Amphinome rostrata*	2.90%	Mobile	Sexual	Internal/External	Planktonic	Carnivore	Predator	Donlan & Nelson 2003 [1]
Mollusca	*Fiona pinnata*	1.90%	Mobile	Sexual	Internal	Planktonic	Carnivore	Predator	Bieri 1966 [84]; Holleman 1972 [86]; Trickey 2013 [87]
Bryozoa	*Arbopercula angulata*	1.00%	Sessile	Sexual/Asexual	Internal	Planktonic	Herbivore	Suspension feeder	Bock 1982 [BRYOZOA] [70]; Weaver et al. 2018 [BRYOZOA] [71]

Supplementary Table 2. Biogeography and taxonomy of rafted taxa.

Column labels are defined as follows: Taxon = species or taxon name (+, not found on JTMD); Probable Biogeographic Origin of Rafting Populations = Biogeographic region where taxa is native (NWP = Northwest Pacific Ocean; NEP = Northeast Pacific Ocean; SIO = Southern Indian Ocean; * = presumptive origin, see Methods); Notes and References = General notes about taxa and literature references (JTMD = Japanese tsunami marine debris)

Taxon	Probable Biogeographic Origin of Rafting Populations	Notes and References
**PORIFERA (sponges)**		
?*Haliclona* sp. A	*NWP	Mat-forming white encrusting species.
?*Leucosolenia* sp.	*NWP	*Leucosolenia eleanor* Urban, 1906 on JTMD [88]; white, branching, encrusting. Amphipacific.
?*Sycon* sp.	*NWP	*Sycon* spp. on JTMD [88]. Erect white vase sponge. Amphipacific taxa.
?*Halichondria* sp.	*NWP	*Halichondria* spp. on JTMD [88]. Yellow and orange encrusting mat. Amphipacific taxa.
**CNIDARIA (hydroids and sea anemones)**		
Aglaophenia aff. *pluma*	NWP	On JTMD [22]; NEP records require confirmation [32].
Plumularia strictocarpa	NWP	*Plumularia* spp. on JTMD [88]. *P. strictocarpa c* an be difficult to distinguish from *P. setacea* without gonothecae. Distribution in North Atlantic associated with pelagic *Sargassum* . Reported from Japan [80].
Clytia hemisphaerica	*NWP	On JTMD [22]. May be a species complex. Amphipacific taxa.
+*Antennella secundaria*	NWP	Not found on JTMD [22, 80]
Diadumene lineata	NWP	On JTMD [22, 89]. Introduced to NEP.
Anthopleura sp. A	NWP	Molecular and morphological study suggest affinities to Northwest Pacific *Anthopleura* sp. Clade J as described in Daly et al. (2017) [90]. Does not match any known NEP anemone. Black/purple body with vertical lines of white verrucae on column.
Anthopleura sp. B	NWP	Molecular and morphological study suggest affinities to Northwest Pacific *Anthopleura* sp. Clade J as described in Daly et al. (2017) [90]. Does not match any known NEP anemone. Tan with vertical lines of grey/purple verrucae along column. Morphology matches *Anthopleura* on JTMD [22].
+*Anemonia erythraea*	NWP/SIO	Molecular data attribute this species to *Anemonia erythraea* in Clade SA with origins in the Southern Indian Ocean as described in Daly et al. (2017) [90]. However, *A. erythraea* is also described from Japan [91]. Smooth, tan body column lacking prominent verrucae found in *Anthopleura* sp. A and *Anthopleura* sp. B.
Anthopleura sp. D	NWP	Molecular and morphological study suggest affinities to Northwest Pacific *Anthopleura* sp. Clades J and A as described in Daly et al. (2017) [90]. A large anemone with mottled brown column lacking prominent verrucae found in *Anthopleura* sp. A and *Anthopleura* sp. B; white stripes along pedal disc.
**ANNELIDA (polychaete worms)**		
Spirorbidae sp.	*NWP	On JTMD [22]
Hydroides sp. cf. *ezoensis*	NWP	On JTMD [22]
Nereididae sp.	*NWP	Family represented on JTMD [22]
**ARTHROPODA (crustaceans and sea spiders)**		
Stenothoe gallensis	NWP	On JTMD as *Stenothoe crenulata*- complex [22, 92].
+*Elasmopus rapax*	NWP	Not found on JTMD [22, 92]. Introduced to NEP.
Jassa marmorata	NWP	On JTMD [22]. North Atlantic native, introduced to NEP.
Calliopius pacificus	*NWP	On JTMD (Carlton, unpublished). Equal antennae. No telson. Amphipacific taxa.
+Amphilochidae sp.	*NWP	Not found on JTMD [22]. Equal, short antennae. Rounded telson. Amphipacific taxa.
Ianiropsis serricaudis	NWP	On JTMD [22]. Introduced to NEP [94].
Endeis nodosa	NWP	On JTMD [22, 94]. Adults; juvenile *Endeis* sp. also present.
**MOLLUSCA (bivalves)**		
Crassostrea gigas	NWP	On JTMD [22]. Introduced to NEP.
Musculus cupreus	NWP	On JTMD [22]
**BRYOZOA**		
Crisia sp.	NWP	*Crisia* spp. on JTMD [22, 95]
Disporella sp.	*NWP	*Disporella cf. novaehollandiae* on JTMD [22, 95]
?*Tubulipora* sp.	NWP	*Tubulipora* spp. on JTMD [22, 95]
Scruparia ambigua	NWP	On JTMD [22, 95]
Catenicella sp.	NWP	On JTMD [22, 95]
+*Aetea* sp. A	*NWP	A corrugated stalk *Aetea* not found on JTMD [22, 95]
+*Aetea* sp. B	*NWP	Not found on JTMD [22, 95]. NWP Aetidae are poorly known (a previously undetected genus in the NWP, *Callaetea* , being discovered on JTMD). *Aetea* sp. B appears reminiscent of NEP *A. pseudoanguina* [96], but our material consists of only 3 zooids.
Aetea anguina	NWP	On JTMD [22, 95]
Callaetea sp.	NWP	On JTMD, apparently an undescribed species [22, 95]
Bugula tsunamiensis	NWP	On JTMD [22, 95]
Cryptosula pallasiana	NWP	On JTMD [22, 95] Introduced to NEP.
+*Amathia gracilis*	*NWP	(= *Bowerbankia gracilis*). Not found on JTMD [22, 95]

References

67. Ambrose, R.F. & Anderson, T.W. Influence of an artificial reef on the surrounding infaunal community. *Mar. Biol.* **107**, 41-52 (1990).
68. Svoboda, A. & Cornelius, P.F.S. The European and Mediterranean species of Aglaophenia (Cnidaria: Hydrozoa). (Nationaal Natuurhistorisch Museum, 1991).
69. Macdonald, T.A., Burd, B.J., Macdonald, V.I., & van Roodselaar, A. Taxonomic and feeding guild classification for the marine benthic macroinvertebrates of the Strait of Georgia, British Columbia (Canadian Technical Report of Fisheries and Aquatic Sciences 2874, 2010).
70. Bock, P.E. Bryozoans (Phylum Bryozoa). in *Marine Invertebrates of Southern Australia* , Part 1, 319-394. (South Australian Government, Adelaide, 1982).
71. Weaver, H., Cook, P., Bock, P. & Gordon, D. *Australian Bryozoa* , Vol 2: Taxonomy of Australian Families. (Csiro Publishing, 2018).
72. Ferreira, D.R.J. et al. Temporal variation in peracarid assemblages inhabiting Caulerpa racemosa in two Brazilian rocky shores. Marine Biodivers. **49**, 1253-1260 (2019).
73. Bocharova, E.S. & Kozevich, I.A. Modes of reproduction in sea anemones (Cnidaria, Anthozoa). Biol. Bull. **38**, 849-860 (2011).
74. Terlizzi, A., Conte, E. & Giangrande, A. Settlement patterns of two Spirorbidae (Annelida, Polychaeta) species in the harbour of Ischia (Gulf of Naples, Mediterranean Sea). Ital. J. Zool. **67**, 303-306 (2000).
75. Takeda, N. et al. Identification of jellyfish neuropeptides that act directly as oocyte maturation-inducing hormones. Development **145**, dev15678 (2018).
76. Maldonado, M. & Riesgo A. Reproduction in the phylum Porifera: a synoptic overview. Treballs de la SCB. **59**, 29-49 (2008).
77. Hirohito, E.S. *The Hydroids of Sagami Bay* . Part II. Thecata. 335 (Biological Laboratory, Imperial Household, Tokyo, Japan, 1995).
78. Kozloff, E. N. *Invertebrates* . (Saunders College Publishing, 1990).
79. Reed, C. G. The reproductive biology of the gymnolaemate bryozoan Bowerbankia gracilis (Ctenostomata: Vesiculariidae). Ophelia. **29**, 1-23 (1988).
80. Guerra-Garcia, J.M et al. Dietary analysis of the marine Amphipoda (Crustacea: Peracardia) from the Iberian Peninsula. J. Sea. Res. **85**, 508-517 (2014).
81. Taylor, P.D. & Monks, N. A new cheilostome bryozoan genus pseudoplanktonic on molluscs and algae. Invert. Biol. **116**, 39-51 (1997).
82. Howard, G.K. & Scott, H.C. Predaceous feeding in two common gooseneck barnacles. Science. **129**, 717-718 (1959).
83. Patel, B. The influence of temperature on the reproduction and moulting of Lepas anatifera L. under laboratory conditions. J. Mar. Biol. Assoc. U.K. **38**, 589-597 (1959).
84. Bieri, R. Feeding preferences and rates of the snail, Ianthina prolongata, the barnacle, Lepas anserifera, the nudibranchs, Glaucus atlanticus and Fiona pinnata, and the food web in the marine neuston. Pub. Seto Mar. Biol. Lab. **14**, 161-170 (1966).
85. Moyse, J. Larvae of lepadomorph barnacles. in *Barnacle Biology* . 329-362 (1987).
86. Holleman, J.J. Observations on growth, feeding, reproduction, and development in the opisthobranch Fiona pinnata (Eschscholtz). Veliger. **15**, 142-146 (1972).
87. Trickey, J. Phylogeography and molecular systematics of the rafting aeolid nudibranch Fiona pinnata (Eschscholtz, 1831) (Doctoral dissertation, University of Otago, 2013).

88. Elvin, D.W. et al. Porifera (Sponges) from Japanese Tsunami marine debris arriving in the Hawaiian Islands and on the Pacific coast of North America. Aquat. Inv. 13, 31-41 (2018).
89. Glon, H. et al. Mediators of invasions in the sea: life history strategies and dispersal vectors facilitating global sea anemone introductions. Biol. Invasions. **22**, 3195–3222 (2020).
90. Daly, M. et al. Anthopleura and the phylogeny of Actinioidea (Cnidaria: Anthozoa: Actiniaria). Org. Divers. Evol. **17**, 545–564 (2017).
91. Shiomi, K. et al. Novel polypeptide toxins with crab lethality from the sea anemone Anemonia erythraea. Biochim. Biophys. Acta **1335**, 191-198 (1997).
92. Carlton, J.T. & Eldredge, L.G. Marine Bioinvasions of Hawaiʻi. (Bishop Museum/Bishop Museum Press, 2009).
93. Hobbs, N.V. et al. Going global: the introduction of the Asian isopod Ianiropsis serricaudis Gurjanova (Crustacea: Peracarida) to North America and Europe. Aquat. Inv. **10**, 177-187 (2015).
94. Nakamura, K. & Child, C.A. Pycnogonida from waters adjacent to Japan. Smithson. Contr. Zool. **512**, 35 (1991).
95. McCuller, M.I. & Carlton, J.T. Transoceanic rafting of Bryozoa (Cyclostomata, Cheilostomata, and Ctenostomata) across the North Pacific Ocean on Japanese tsunami marine debris. Aquat. Inv. **13**, 137-162 (2018).
96. Soule, D.F., Soule, J.D. & H.W. Chaney. *Taxonomic atlas of the benthic fauna of the Santa Maria Basin and western Santa Barbara Channel* . (Santa Barbara Museum of Natural History, 1995).
