# Supplementary material for: Extent and reproduction of coastal species on plastic debris in the North Pacific Subtropical Gyre
Source: Nat Ecol Evol. 2023 Apr 17;7(5):687–97. doi: 10.1038/s41559-023-01997-y (PMC10172146; doi:10.1038/s41559-023-01997-y)
Supplement: Supplementary file 2 — Reporting Summary [file 41559_2023_1997_MOESM2_ESM.pdf]

nature portfolio

Corresponding author(s): Linsey Haram

Last updated by author(s): Nov 2, 2022

Reporting Summary

Nature Portfolio wishes to improve the reproducibility of the work that we publish. This form provides structure for consistency and transparency in reporting. For further information on Nature Portfolio policies, see our Editorial Policies and the Editorial Policy Checklist.

Statistics

For all statistical analyses, confirm that the following items are present in the figure legend, table legend, main text, or Methods section.

n/a	Confirmed	
☐	☒	The exact sample size (*n*) for each experimental group/condition, given as a discrete number and unit of measurement
☐	☒	A statement on whether measurements were taken from distinct samples or whether the same sample was measured repeatedly
☐	☒	The statistical test(s) used AND whether they are one- or two-sided<br>*Only common tests should be described solely by name; describe more complex techniques in the Methods section.*
☐	☒	A description of all covariates tested
☐	☒	A description of any assumptions or corrections, such as tests of normality and adjustment for multiple comparisons
☐	☒	A full description of the statistical parameters including central tendency (e.g. means) or other basic estimates (e.g. regression coefficient) AND variation (e.g. standard deviation) or associated estimates of uncertainty (e.g. confidence intervals)
☐	☒	For null hypothesis testing, the test statistic (e.g. *F*, *t*, *r*) with confidence intervals, effect sizes, degrees of freedom and *P* value noted<br>*Give P values as exact values whenever suitable.*
☐	☒	For Bayesian analysis, information on the choice of priors and Markov chain Monte Carlo settings
☐	☒	For hierarchical and complex designs, identification of the appropriate level for tests and full reporting of outcomes
☒	☐	Estimates of effect sizes (e.g. Cohen's *d*, Pearson's *r*), indicating how they were calculated

Our web collection on statistics for biologists contains articles on many of the points above.

Software and code

Policy information about availability of computer code

Data collection	Analyses were conducted using standard methods for the statistical packages used in R.
Data analysis	R Studio

For manuscripts utilizing custom algorithms or software that are central to the research but not yet described in published literature, software must be made available to editors and reviewers. We strongly encourage code deposition in a community repository (e.g. GitHub). See the Nature Portfolio guidelines for submitting code & software for further information.

Data

Policy information about availability of data

All manuscripts must include a data availability statement. This statement should provide the following information, where applicable:

- Accession codes, unique identifiers, or web links for publicly available datasets
- A description of any restrictions on data availability
- For clinical datasets or third party data, please ensure that the statement adheres to our policy

The data for this research are available upon publication at the Dryad data depository indicated in the manuscript. https://doi.org/10.5061/dryad.k98sf7m9d

Human research participants

Policy information about studies involving human research participants and Sex and Gender in Research.

Reporting on sex and gender	The present study did not involved human research participants.
Population characteristics	*Describe the covariate-relevant population characteristics of the human research participants (e.g. age, genotypic information, past and current diagnosis and treatment categories). If you filled out the behavioural & social sciences study design questions and have nothing to add here, write "See above."*
Recruitment	*Describe how participants were recruited. Outline any potential self-selection bias or other biases that may be present and how these are likely to impact results.*
Ethics oversight	*Identify the organization(s) that approved the study protocol.*

Note that full information on the approval of the study protocol must also be provided in the manuscript.

Field-specific reporting

Please select the one below that is the best fit for your research. If you are not sure, read the appropriate sections before making your selection.

☐ Life sciences ☐ Behavioural & social sciences ☒ Ecological, evolutionary & environmental sciences

For a reference copy of the document with all sections, see nature.com/documents/nr-reporting-summary-flat.pdf

Ecological, evolutionary & environmental sciences study design

All studies must disclose on these points even when the disclosure is negative.

Study description	This study took place as part of a larger research program known as Floating Ocean Ecosystems (FloatEco), which was funded by the National Aeronautics and Space Administration to investigate the biological and physical underpinnings of the ENPSG through interdisciplinary research and participation of non-profit and volunteer partners, who aided in debris collection (see www.floateco.org for more information about the FloatEco project). For the research presented here, the non-profit The Ocean Cleanup facilitated collection of floating plastic debris during two expeditions aboard Maersk Transporter in November 2018 and January 2019.
Research sample	Crewmembers collected floating plastic debris greater than 15 cm in length, width, or height.
Sampling strategy	Because of variation in debris type in the ENPSG, we standardized collections by targeting items in 10 pre-designated categories based on what is most encountered in that system41 (L. Lebreton, personal observation): bottles, buoys, fish crates, eel or hagfish trap cones, plastic fragments, miscellaneous household items (such as clothes hangers and flowerpots), jugs/buckets, fishing nets, rope, and “wildcard items” (Supplementary Fig. S2). Wildcard items were items that stood out to observers for their density and diversity of attached biota and/or did not fit into the other 9 designated categories. In total, approximately 10 items were collected within each category (Fig. S1). To assess biota frequency on ENPSG debris, items from all categories except the wildcard category were collected and sampled as they were encountered. For example, the first 10 fishing nets encountered were collected and sampled regardless of evidence of attached biota. We did this to avoid sampling bias toward biota-rich debris. Wildcard items were collected opportunistically throughout the survey period.
Data collection	Upon collection, each item was given a unique identifying number and the latitude/longitude, date and time, and debris characteristics (type, size category) were recorded by shipboard personnel. Subsequently, the item was photographed from all sides and it was noted if biota discernible to the naked eye were attached. If biota appeared to be absent, the item was placed in a plastic bag and frozen or dried on deck if too large for the freezer. If biota were present, either approximately 5 individuals of each recognizable taxon (morphotype) were removed and preserved in 95% non-denatured ethanol or the entire debris item was frozen for later sampling in the lab. Sampled debris items were placed in individual bags and frozen or dried on the deck. Preserved (frozen, ethanol, and dried) items were returned to shore and analyzed as follows. Identifying marks (manufacturer names, logos, numbers, or locations) if present were recorded. All items were classified into size categories, defined as small (20-50 cm), medium (50-500 cm), or large (> 500 cm) (see Supplementary Table S1), and examined in a standardized search effort of 15 minutes for macroscopic species, the smaller of which were then examined using a dissecting microscope as needed, outside of the 15 minute search effort. In addition, all frozen and ethanol organisms were preserved or re-preserved in 70% non-denatured ethanol. Voucher specimens from this study are deposited at the Smithsonian Environmental Research Center in Edgewater, Maryland and the Royal BC Museum in Victoria, British Columbia.
Timing and spatial scale	There were two sampling periods: the two sampling periods: November 12-22, 2018 and December 16, 2018 - January 3, 2019.
Data exclusions	No data were excluded from the analyses.
Reproducibility	No experiments were conducted.

Randomization	All samples collected were analyzed.
Blinding	All samples collected were analyzed; no blinding was required.

Did the study involve field work? ☒ Yes ☐ No

Field work, collection and transport

Field conditions	Ocean-going vessels sailed onto the high seas to collect samples.
Location	Collections took place in the Eastern North Pacific Ocean Subtropical Gyre (ENPSG) midway between the coasts of California and Hawaii (Supplementary Fig. S1). The North Pacific Subtropical Gyre is an area of convergence encircled by the Kuroshio, North Pacific, California, and North Equatorial Currents. In the eastern part of the North Pacific Subtropical Gyre (east of the dateline), converging surface currents collect floating debris.
Access & import/export	The samples were collected on the high seas beyond any national jurisdiction. No import-export permits or other permits were required in this work.
Disturbance	No disturbances were caused or created by this study; by removing plastic, we were helping to minimize or remove disturbance.

Reporting for specific materials, systems and methods

We require information from authors about some types of materials, experimental systems and methods used in many studies. Here, indicate whether each material, system or method listed is relevant to your study. If you are not sure if a list item applies to your research, read the appropriate section before selecting a response.

Materials & experimental systems

n/a	Involved in the study	
☒	☐	Antibodies
☒	☐	Eukaryotic cell lines
☒	☐	Palaeontology and archaeology
☐	☒	Animals and other organisms
☒	☐	Clinical data
☒	☐	Dual use research of concern

Methods

n/a	Involved in the study	
☒	☐	ChIP-seq
☒	☐	Flow cytometry
☒	☐	MRI-based neuroimaging

Animals and other research organisms

Policy information about studies involving animals; ARRIVE guidelines recommended for reporting animal research, and Sex and Gender in Research

Laboratory animals	No laboratory animals were involved.
Wild animals	The study did not involve wild animals.
Reporting on sex	Not applicable.
Field-collected samples	If invertebrate biota were present, either approximately 5 individuals of each recognizable taxon (morphotype) were removed and preserved in 95% non-denatured ethanol or the entire debris item was frozen for later sampling in the lab. Sampled debris items were placed in individual bags and frozen or dried on the deck. Preserved (frozen, ethanol, and dried) items were returned to shore and analyzed as follows. Identifying marks (manufacturer names, logos, numbers, or locations) if present were recorded. All items were classified into size categories, defined as small (20-50 cm), medium (50-500 cm), or large (> 500 cm) (see Supplementary Table S1), and examined in a standardized search effort of 15 minutes for macroscopic species, the smaller of which were then examined using a dissecting microscope as needed, outside of the 15 minute search effort. In addition, all frozen and ethanol organisms were preserved or re-preserved in 70% non-denatured ethanol. Voucher specimens from this study are deposited at the Smithsonian Environmental Research Center in Edgewater, Maryland and the Royal BC Museum in Victoria, British Columbia.
Ethics oversight	No ethical approval or guidance was required. No human subjects were involved and no animals were subjected to field or laboratory experiments.

Note that full information on the approval of the study protocol must also be provided in the manuscript.
